# Computer-Assisted Orthognathic Surgery for Patients with Cleft Lip/Palate: From Traditional Planning to Three-Dimensional Surgical Simulation

**DOI:** 10.1371/journal.pone.0152014

**Published:** 2016-03-22

**Authors:** Daniel Lonic, Betty Chien-Jung Pai, Kazuaki Yamaguchi, Peerasak Chortrakarnkij, Hsiu-Hsia Lin, Lun-Jou Lo

**Affiliations:** 1 Department of Plastic and Reconstructive Surgery and Craniofacial Research Center, Chang Gung Memorial Hospital, Chang Gung University, Taoyuan, Taiwan; 2 Department of Craniofacial Orthodontics, and Craniofacial Research Center, Chang Gung Memorial Hospital, Chang Gung University, Taoyuan, Taiwan; 3 Division of Plastic Surgery, Siriraj Hospital, Mahidol University, Bangkok, Thailand; 4 Craniofacial Research Center, Chang Gung Memorial Hospital, Taoyuan, Taiwan; Medical University of South Carolina, UNITED STATES

## Abstract

**Background:**

Although conventional two-dimensional (2D) methods for orthognathic surgery planning are still popular, the use of three-dimensional (3D) simulation is steadily increasing. In facial asymmetry cases such as in cleft lip/palate patients, the additional information can dramatically improve planning accuracy and outcome. The purpose of this study is to investigate which parameters are changed most frequently in transferring a traditional 2D plan to 3D simulation, and what planning parameters can be better adjusted by this method.

**Patients and Methods:**

This prospective study enrolled 30 consecutive patients with cleft lip and/or cleft palate (mean age 18.6±2.9 years, range 15 to 32 years). All patients received two-jaw single-splint orthognathic surgery. 2D orthodontic surgery plans were transferred into a 3D setting. Severe bony collisions in the ramus area after 2D plan transfer were noted. The position of the maxillo-mandibular complex was evaluated and eventually adjusted. Position changes of roll, midline, pitch, yaw, genioplasty and their frequency within the patient group were recorded as an alternation of the initial 2D plan. Patients were divided in groups of no change from the original 2D plan and changes in one, two, three and four of the aforementioned parameters as well as subgroups of unilateral, bilateral cleft lip/palate and isolated cleft palate cases. Postoperative OQLQ scores were obtained for 20 patients who finished orthodontic treatment.

**Results:**

83.3% of 2D plans were modified, mostly concerning yaw (63.3%) and midline (36.7%) adjustments. Yaw adjustments had the highest mean values in total and in all subgroups. Severe bony collisions as a result of 2D planning were seen in 46.7% of patients. Possible asymmetry was regularly foreseen and corrected in the 3D simulation.

**Conclusion:**

Based on our findings, 3D simulation renders important information for accurate planning in complex cleft lip/palate cases involving facial asymmetry that is regularly missed in conventional 2D planning.

## Introduction

Orthognathic surgery (OGS) is a powerful procedure for reshaping the osseous structures of the midface and mandible in patients with congenital or developmental dentofacial deformities [1]. These changes also affect facial soft tissues and can significantly improve overall facial appearance. Achieving a successful outcome requires precise and safe surgical technique, appropriate orthodontic management and, maybe most importantly, accurate preoperative planning to ideally align the bone segments [2, 3]. Surgical design should not only consider skeletal and dental aspects, but also harmonize the soft tissue, profile and overall facial appearance.

The majority of OGS procedures are still planned using traditional two-dimensional (2D) cephalometry; nontheless, three-dimensional (3D) simulation in OGS planning has gained increasing popularity [4]. The application of 3D planning enables the surgeon to virtually simulate the surgery before entering the operating room [5]. Because the benefits of 3D simulation and its distribution of use are still discordant, a reevaluation of the possibilities and limitations of both 2D and 3D methods is needed [6]. Numerous studies have demonstrated the feasibility and accuracy of 3D simulation [4, 7–17], and the increasing use in OGS planning has lead to significant outcome improvement [18]. On the other hand, more information has to be processed when planning OGS in three dimensions, especially in cases involving facial asymmetry such as in patients with cleft lip/palate (CL/P)[12, 19].

When OGS planning is carried out using 2D cephalometry, both occlusal and skeletal relationships have to be adressed. However, if 2D cephalometry planning is transferred into a 3D simulation environment, previously undetected problems can show up as pitch, roll and yaw discrepancies, severe bony collisions in the ramus area, genioplasty malposition, and midline deviation. These issues have to be corrected respecting facial symmetry, harmony and soft tissue appearance, and the initial 2D treatment plan should be altered accordingly in agreement with the orthodontist.

Although the “normal” face is almost always asymmetrical [20, 21] to some degree, asymmetry in its exaggerated form is the leading feature in CL/P patients [22, 23], more so in the unilateral, but also in the bilateral and isolated cleft palate cases. Concomitant malocclusion and pathological dentition are ubiquitous in these cases, and facial asymmetry makes OGS planning especially demanding. Malocclusion, bony deficiencies and facial asymmetry all have to be addressed in the surgical plan to yield an optimal result.

The purpose of this study is to investigate which parameters are changed most frequently for improvement of OGS planning when a traditional 2D plan is transferred to 3D simulation, and how extensive these changes are in terms of angular or linear measurements. Furthermore we try to show if modifying the 2D plan is more the exception or the rule in the preoperative planning process of orthognathic procedures for cleft lip/palate patients with facial deformity and asymmetry.

## Materials and Methods

This prospective study enrolled 30 consecutive CL/P patients from January 2012 through December 2014. The mean age was 18.6±2.9 years, ranging from 15 to 32 years. 20 unilateral CL/P (16 left sided, 4 right sided, mean age 18.0±1.4 years, range 15–20 years, 14 female, 6 male), 6 bilateral CL/P (mean age 18.8±1.2 years, range 17–20 years, 4 female, 2 male) and 4 isolated cleft palate patients (mean age 21.3±7.2 years, range 17–32 years, 3 female, 1 male) were identified in three subgroups. Inclusion criteria were 1) patients planned for single-splint two-jaw OGS, and 2) patients with cone-beam computed tomography (CBCT) scans. Exclusion criteria were the presence of traumatic or syndromic craniofacial deformities. Ethical approval was completed by the Institutional Review Board of Chang Gung Memorial Hospital, Taiwan under IRB 103-2822B. All patients gave written informed consent for using their data in an anonymized fashion. If patients were under 20 years of age, written informed consent was obtained from the parents, next of kin, caretakers, or guardians. Patient records and datasets were anonymized and de-identified prior to analysis.

**Fig 1:**
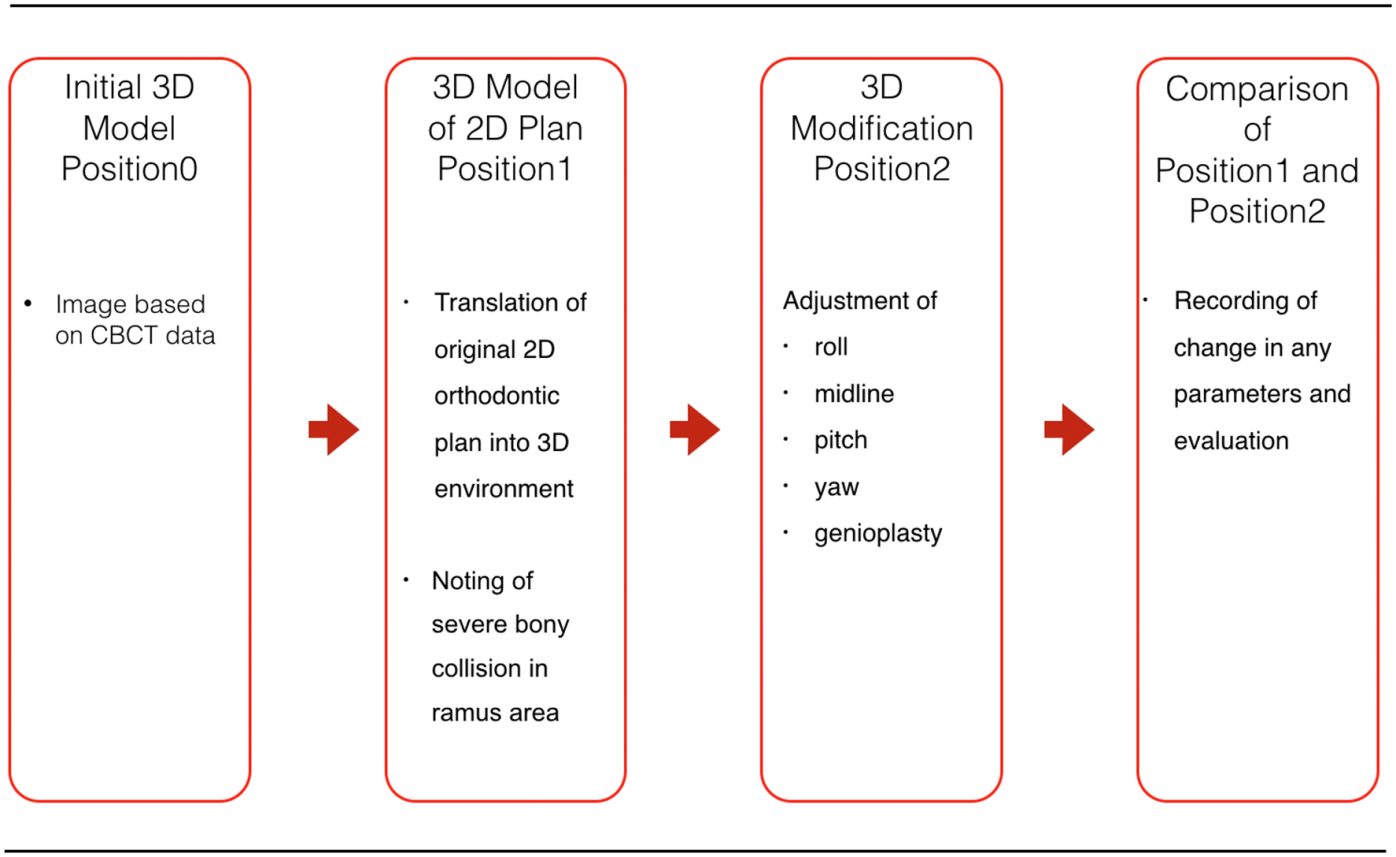
Principles of single-splint two-jaw orthognathic surgery. Both maxilla and mandible are completely osteotomized, fixed in the final dental splint and moved as a united “maxillo-mandibular complex” (MMC) to the desired position. The maxillary segment is then referred to as the Le Fort I segment, and the mandible consists of two proximal (ramus) segments and one distal segment bearing the dental arch and the neurovascular bundle after osteotomy. The rotation movements are described as pitch, roll and yaw, en-bloc linear horizontal movements as left and right shifts or advancements and setbacks in the antero-posterior direction, and en-bloc linear vertical movements as extrusion and intrusion according to the movement in relation to the skull base.

Image scanning and evaluation were conducted in the following sequence (Fig 1). Step 1: After preoperative frontal and lateral cephalograms and photographs, conventional orthodontic OGS planning without articulator setup [24] was carried out using 2D cephalometry. For 3D simulation, CBCT images were obtained for all patients using an i-CAT CBCT scanner (Imaging Sciences International, Hatfield, PA) with a voxel resolution of 0.4 mm in upright position to prevent unwanted gravitational effects on the soft tissue. DICOM files were imported into one of two commercial software programs, Simplant OMS (Materialise, Leuven, Belgium) or Dolphin 3D (Dolphin Imaging & Management Solutions, Chatsworth, California, USA). The initial position of the skeletal structures was recorded as Position0.

**Fig 2:**
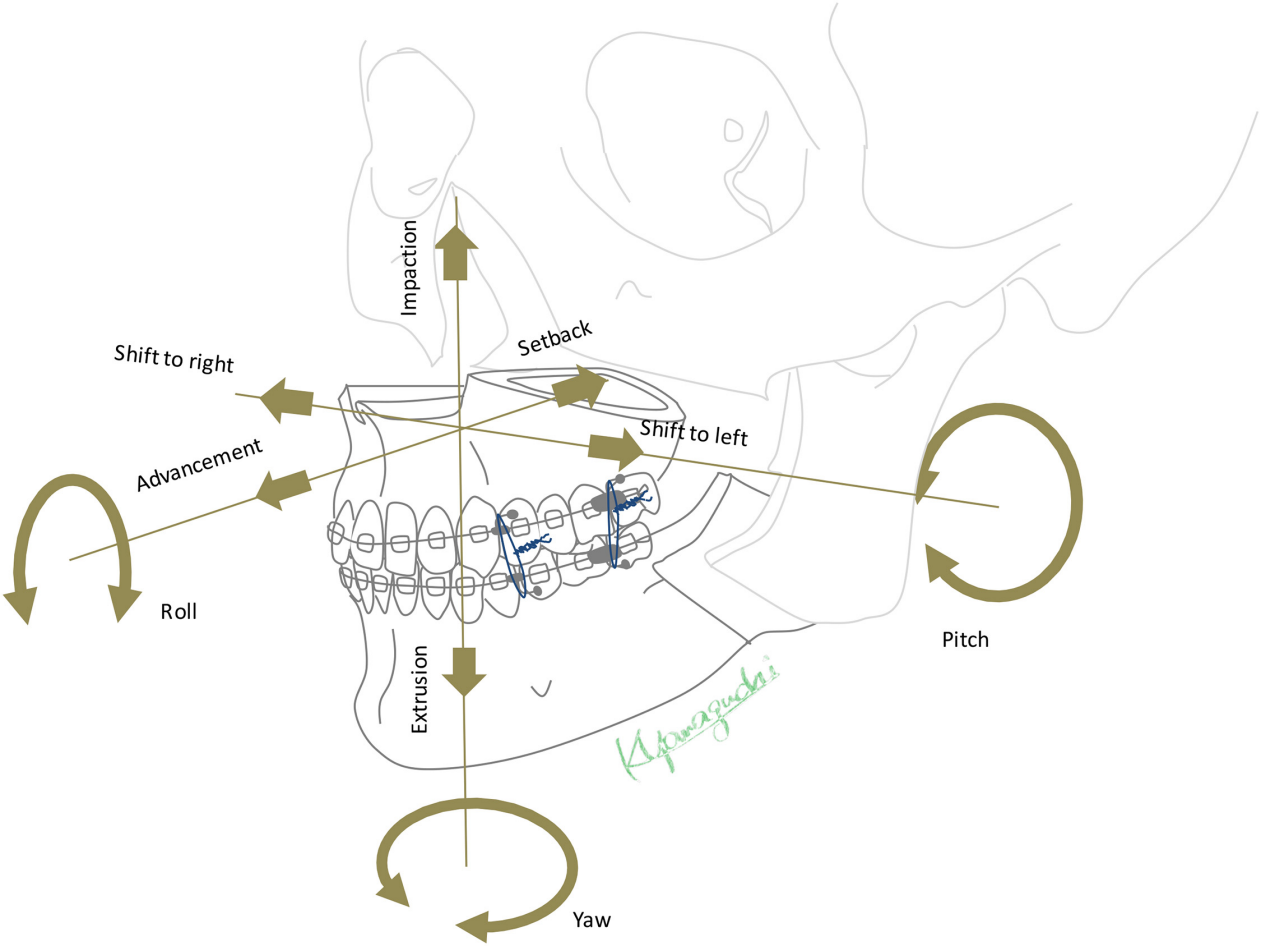
Flowchart of the methodology. In the first step, the patient’s initial preoperative Cone-Beam Computer Tomography (CBCT) defines Position0. After segmentation, the conventional orthodontic 2D plan is transferred into the 3D simulation and recorded as Position1. The transferred plan is then adjusted for roll, midline, pitch, yaw, and genioplasty positions by the surgical team, and the resulting 3D simulation is noted as Position2. The changes in each parameter from Position1 to Position2 are recorded and evaluated.

Step 2: Image segmentation was performed to obtain 3D representation of hard tissues used for 3D simulation. Occlusion was determined from dental plaster models with the dental splint in vitro using a 3Shape Orthodontics System dental surface scanner (Copenhagen, Denmark). The resulting STL files were imported into the respective 3D simulation software [25]. After defining Le Fort I and BSSO osteotomy planes, the maxillary and mandibular segments were aligned according to the plaster model by automatic registration. The artefact-affected dental arches were replaced by plaster model images for a more accurate dental surface [26]. The final occlusion, determined by the occlusal splint, was transferred into the 3D plan by moving the mandible to the fixed maxilla. Subsequently the segments were then moved as one united maxillo-mandibular complex (MMC), maintaining the planned terminal occlusion while resembling the MMC movements during actual single-splint surgery (Fig 2). At this stage, the conventional 2D plan was transferred into the 3D environment by MMC movement according to the orthodontist’s specifications. While some MMC movements (e.g. Le Fort I advancement) could be applied identically, others had to be translated into angular values such as pitch or roll rotation. Linear impaction or extrusion (in millimeters) of the Le Fort I plane at the level of the canine or first molar was used for roll or pitch correction in 2D. Although the MMC movements are angular in 3D Dolphin or Simplant software in the frontal (roll) or right lateral (pitch) view of the patient’s skull, they can also be displayed as linear movements (in millimeters) and matched to the specifications of the 2D plan. The resulting 3D position based on the 2D plan transfer was marked as Position1.

Step 3: During 3D simulation the MMC was moved and rotated by the surgical team until the best position in terms of skeletal harmony, symmetry, facial proportion, teeth exposure in relation to the upper lip, and surgical feasibility was achieved. Midline correction was the utmost concern, followed by facial symmetry. Therefore our simulation sequence started with roll and midline adjustment in the frontal, followed by pitch correction in the lateral and yaw correction in the basal view, with the rotation center in the upper dental midline at the U1 incisor tip level. Bony collisions between proximal and distal mandibular segments were noted and defined as severe if distal segment aspects were lateral to the proximal segment after 2D surgery plan transfer. Proximal and distal mandibular segments were aligned and evaluated for cheek symmetry by removing premature contacts in the ramus area or keeping the inter-segmental space. The surgical team and orthodontist reviewed the final plan and agreed on the MMC position, which was the recorded as as Position2.

**Fig 3:**
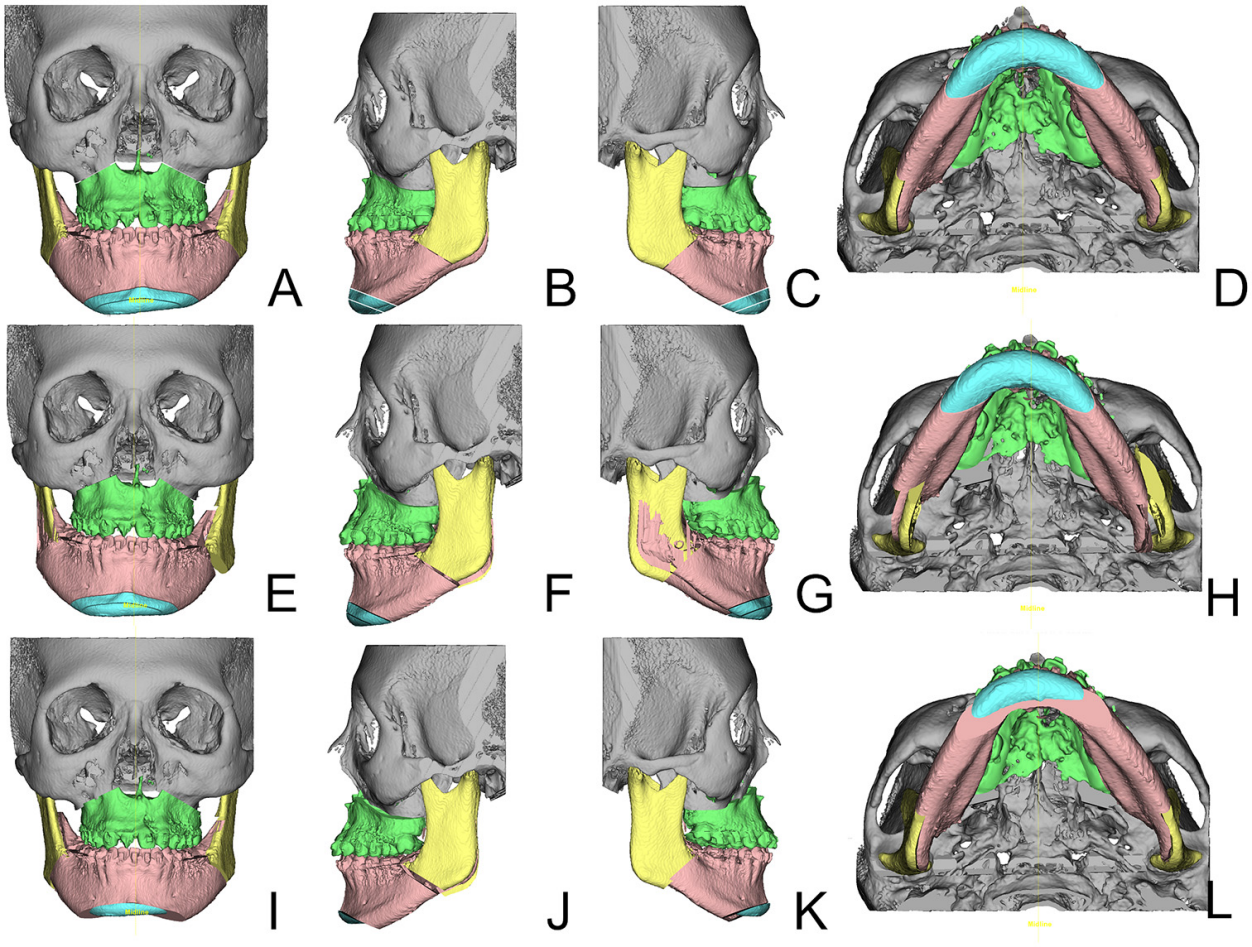
Visualization of the three positions in sample patient #25 (Table 1). Isolated cleft palate patient. From left to right frontal, left lateral, right lateral and basal views are shown of the same position. Subfigures A-D show the initial Position0, Subfigures E-H the result of the 2D plan translation into the 3D environment and Subfigures I-L the adjusted Position2 in the 3D plan agreed upon by the orthodontists and the surgical team. Note the severe bony collision in the right ramus area of this patient in Subfigures E and G and the subsequent large bony gap in the left ramus area. Through counterclockwise yaw rotation the collision and overall asymmetry were resolved (Subfigures H and L, E and I). The genioplasty position was altered by shortening the chin segment, thus reducing the patient’s facial height (Subfigures I-L).

**Fig 4:**
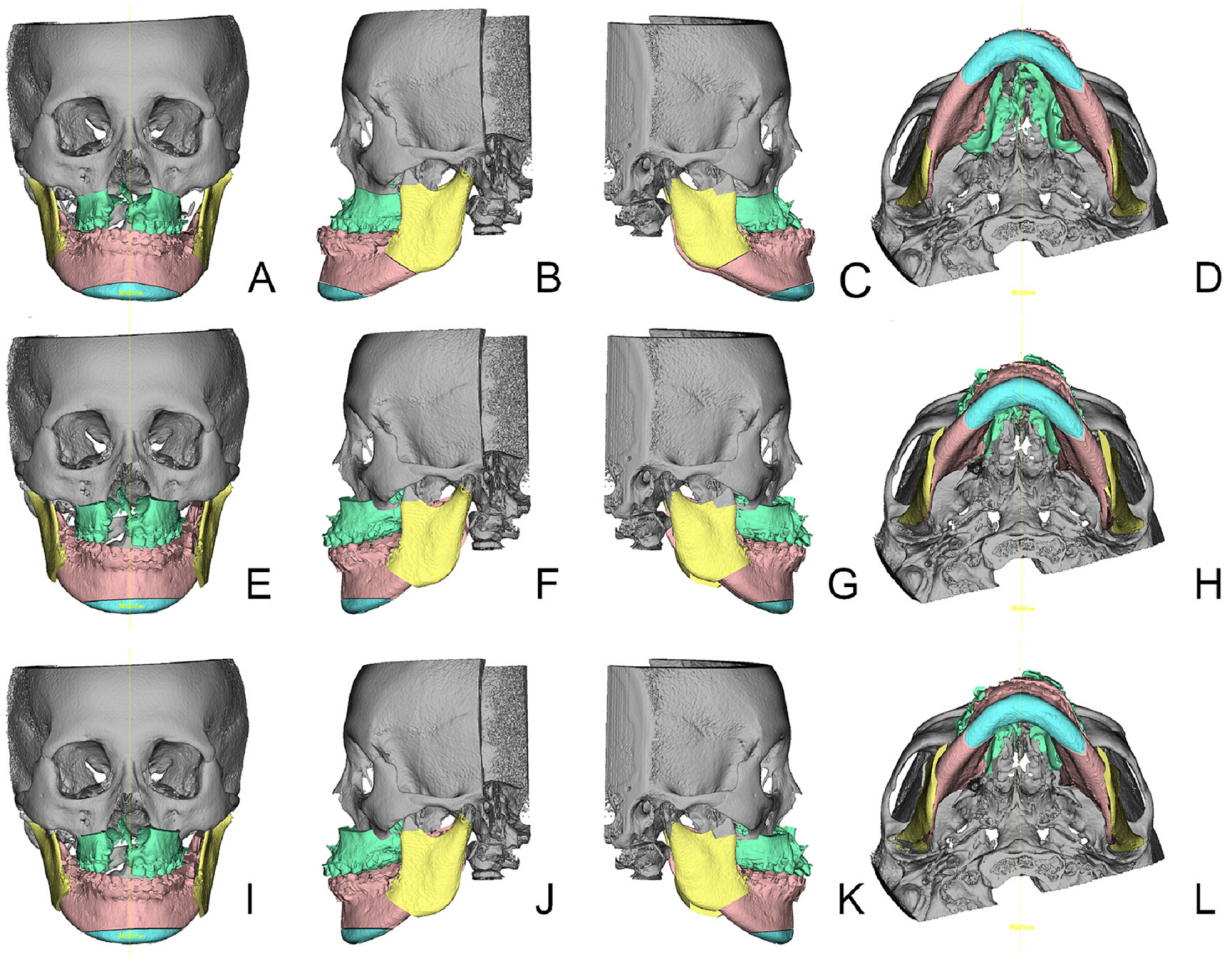
Visualization of the three positions in sample patient #29 (Table 1). Bilateral cleft lip/palate patient. From left to right frontal, left lateral, right lateral and basal views are shown of the same position. Subfigures A-D show the initial Position0, Subfigures E-H the result of the 2D plan translation into 3D environment and Subfigures I-L the adjusted Position2 in 3D simulation agreed upon by the orthodontists and the surgical team. Bony collisions were not seen in this patient, but a yaw correction of 2° counterclockwise seen from basal Subfigures H to L was implemented.

Step 4: As a primary outcome measure, Position1 and Position2 were evaluated in frontal, lateral and basal views with respect to midline, pitch, roll and yaw correction [27]. Furthermore, the need for genioplasty and correction of severe bony collisions for achieving facial harmony were noted. Absolute pitch, roll and yaw corrections were noted in degrees, absolute midline and genioplasty corrections in millimeters. Figs 3 and 4 show the steps from initial Position0 (Figs 3 and 4A–4D) to translated 2D planning Position1 (Figs 3 and 4E–4H) to adjusted 3D planning Position2 (Figs 3 and 4I–4L). Parameter changes were recorded as alternations of the initial 2D plan. Linear midline correction was subdivided into changes less than 1 mm, between 1 to 2 mm and more than 2 mm respectively. Pitch correction of equal to or more than 1mm in the upper first molar Le Fort I level were judged as parameter change. Because of the importance of roll correction for occlusal canting, any change was recorded as a parameter change. Two aspects of yaw correction were recorded; firstly, cases of the aforementioned severe bony collisions in the ramus area were noted. Secondly, to account for possible CBCT imaging errors, patients needing more than one degree of yaw rotation correction were considered having a parameter change. Genioplasty changes were recorded if the position was altered in terms of rotation, impaction, elongation, advancement or general indication. Patients were divided in groups of no change and changes in one, two, three and four parameters. For further analysis, all parameter changes were also investigated within the subgroups of unilateral CL/P, bilateral CL/P and isolated cleft palate patients, and the mean changes, standard deviations and ranges for each parameter were calculated.

**Fig 5:**
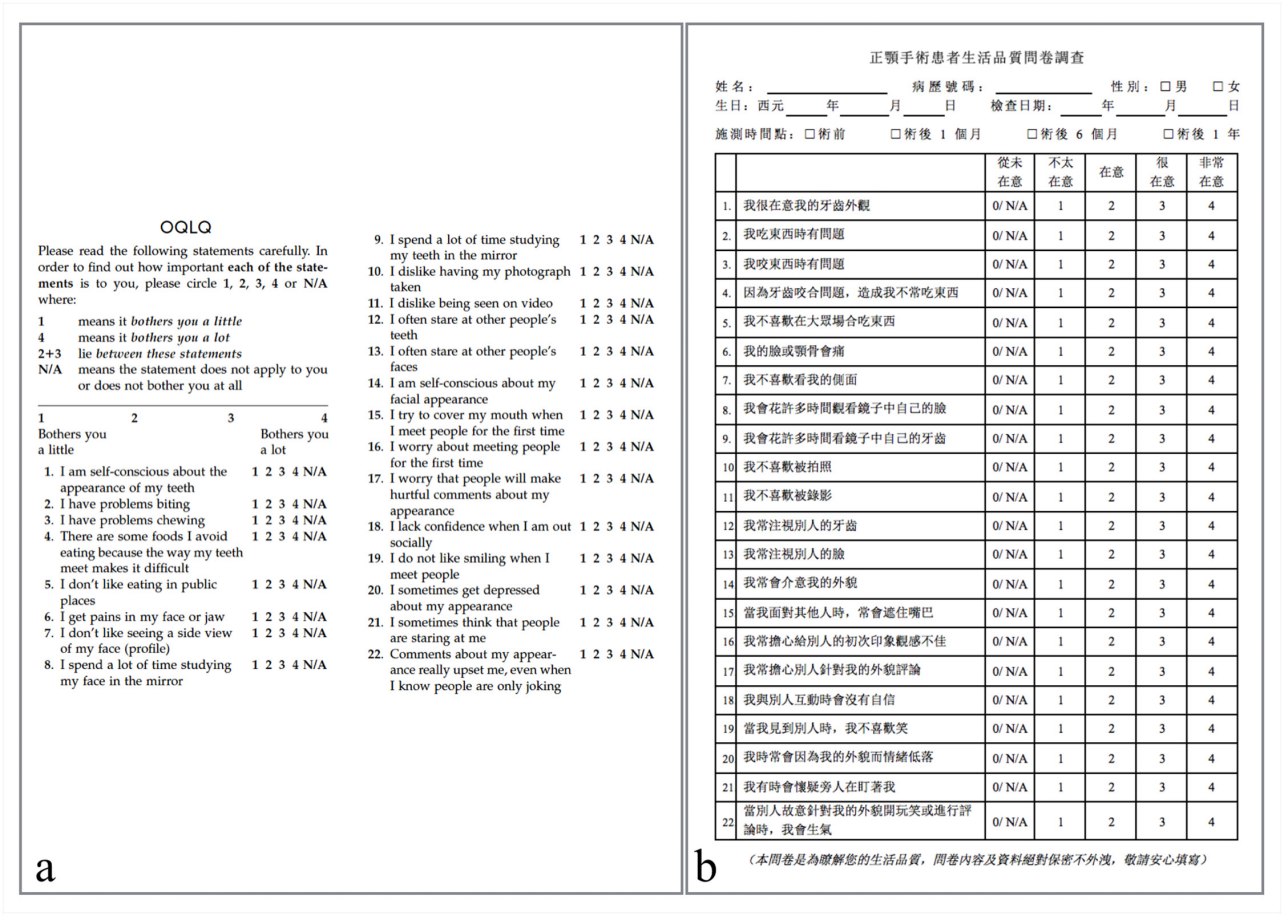
OQLQ questionnaire. English (Fig 5a) and Chinese (Fig 5b) versions of the OQLQ questionnaire measuring the patient’s dentofacial deformity in relation to their quality of life. It consists of a 4-point scale ranging from “bothers you a little” (score 1) to “bothers you a lot” (score 4). If the patient did not feel impaired by the subject (“does not apply, does not bother me”), it was rated as N/A or 0 points. Ranging from 0 to 88, lower OQLQ scores indicate better and higher score indicate poorer quality of life. The 22 items cover 4 topics: facial esthetics (items 1, 7, 10, 11, and 14 scoring 0 to 20), oral function (items 2–6 scoring 0 to 20), awareness of dentofacial esthetics (items 8, 9, 12, and 13) scoring 0 to 16), and social aspects of dentofacial deformity (items 15–22 scoring 0 to 32) [30].

Additionally, patients who finished orthodontic treatment were asked to fill out the OQLQ questionnaire [28, 29] (Fig 5a) in the Chinese translation (Fig 5b) to measure their quality of life in relation to their dentofacial deformity. Ranging from 0 to 88, lower OQLQ scores indicate better and higher scores indicate poorer quality of life. The 22 items cover 4 topics: facial esthetics (scoring 0 to 20), oral function (scoring 0 to 20), awareness of dentofacial esthetics (scoring 0 to 16), and social aspects of dentofacial deformity (scoring 0 to 32) [30]. Means, standard deviations and ranges for total scores and each topic were recorded.

## Results

**Fig 6:**
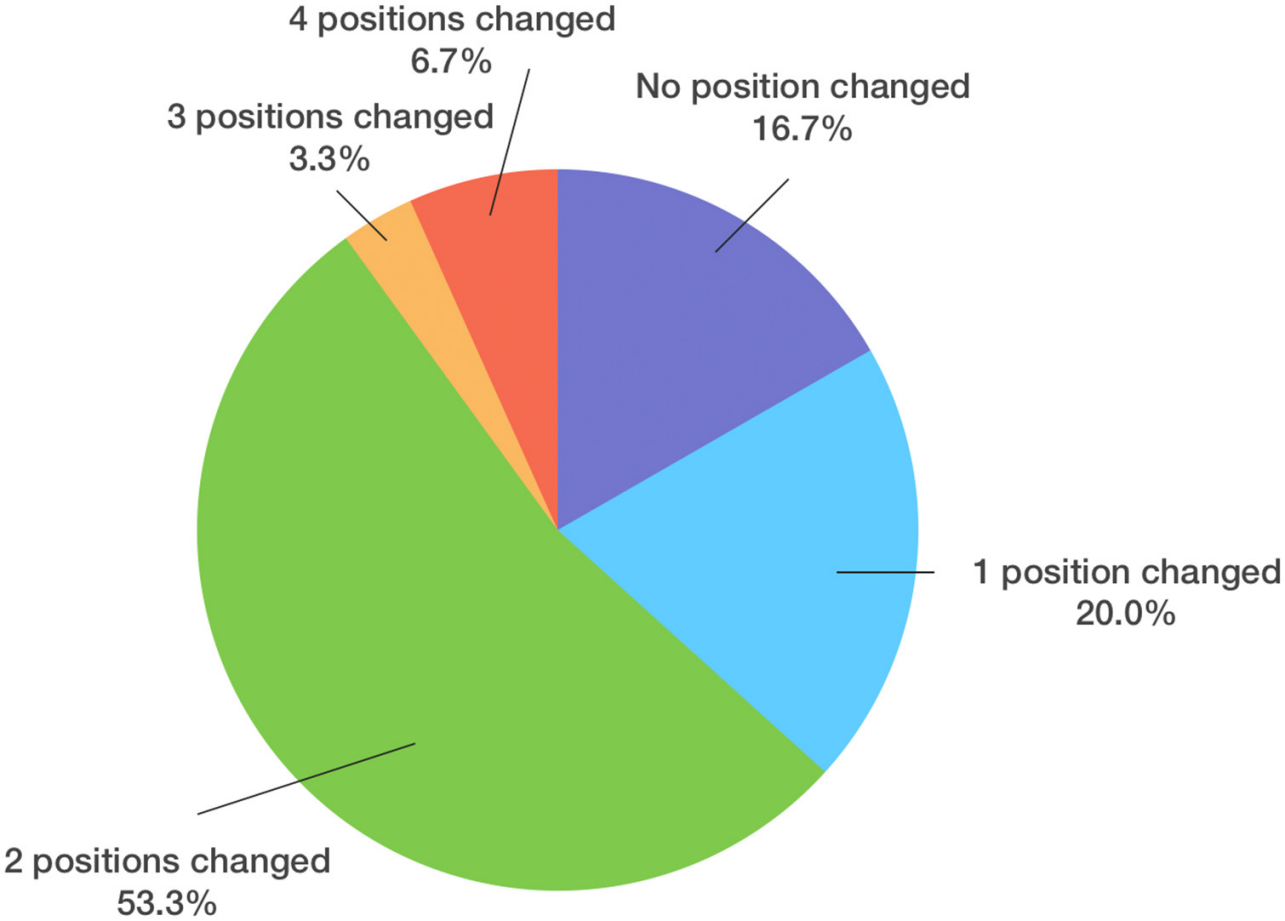
Pie chart of patients with different amounts of position changes. According to the changes from Position1 (2D planning) to Position2 (3D simulation), 5 (16.7%) patients underwent no change, 6 (20.0%) had a change in one, 16 (53.3%) had a change in two, 1 (3.3%) had a change in three and 2 (6.7%) had a change in four parameters (Fig 4). 26 (83.3%) patients underwent the procedure with at least one modification of the initial 2D planning, while 16.7% had no changes from the 2D plan.

**Fig 7:**
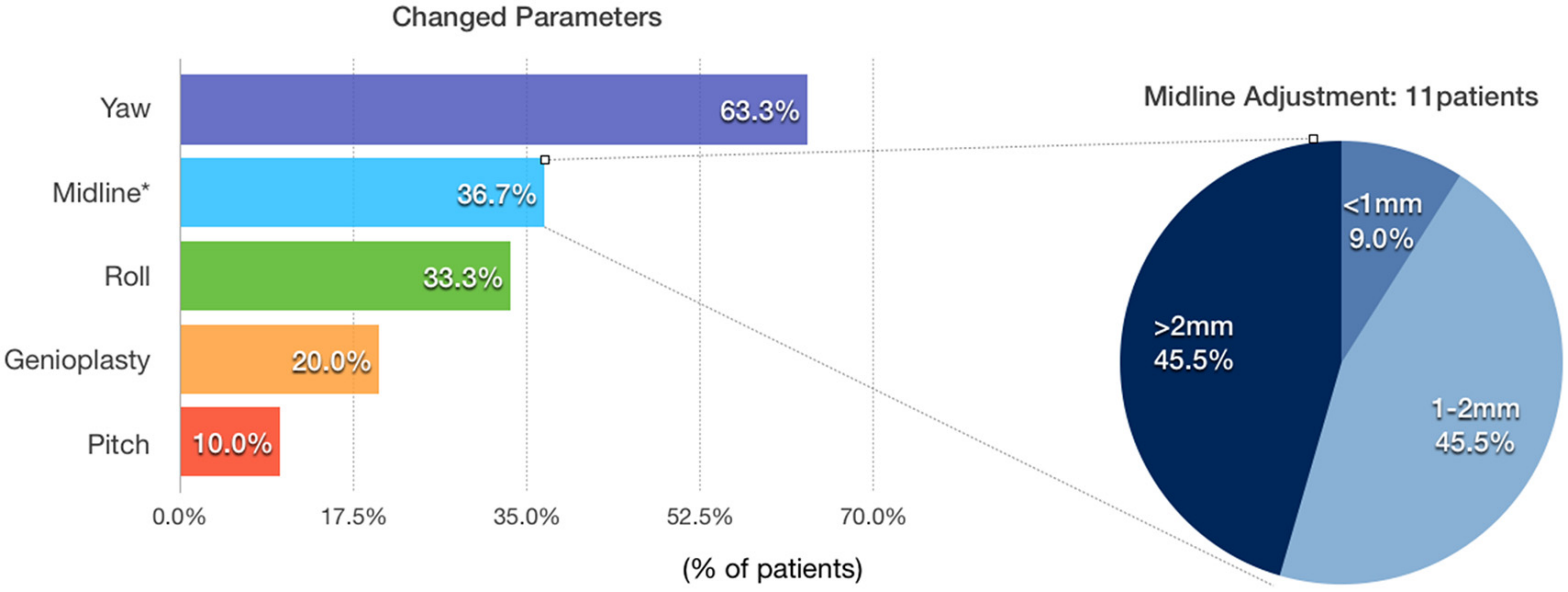
Frequency of parameter changes in the patients. Most frequently changed parameter was yaw rotation (19 patients, 63.3%), followed by midline adjustment (11 patients, 36.7%), roll rotation (9 patients, 33.3%), genioplasty position (6 patients, 20.0%) and pitch rotation (3 patients, 10.0%). Of the 11 patients that had a modification of the midline from 2D to 3D planning, 6 (45.5%) had a movement over 2 mm, 5 (45.5%) between 1 to 2 mm, and 1(9.0%) under 1 mm.

Datasets of 30 consecutive (17 male, 13 female) CL/P patients were evaluated (Table 1). Regarding our primary outcome measure, 5 patients (16.7%) underwent no change, 6 (20.0%) had a change in one, 16 (53.3%) had a change in two, 1 (3.3%) had a change in three and 2 (6.7%) had a change in four parameters (Fig 6). 25 (83.3%) patients underwent the procedure with at least one modification of the initial 2D plan. Yaw was most frequently changed (19 patients, 63.3%, mean 3.0±3.1°, range 0–12°), followed by midline (11 patients, 36.7%, mean 1.0±1.7mm, range 0–6.5mm), roll (10 patients, 33.3%, mean 1.1±2.0°, range 0–7.5°), genioplasty (6 patients, 20.0%, mean 1.0±2.1mm, range 0–6.5mm) and pitch (3 patients, 10.0%, mean 0.3±1.0°, range 0–4°). Five (45.5%) out of 11 patients had a midline movement over 2 mm, 5 (45.5%) between 1 to 2 mm, and 1(9.0%) under 1 mm (Fig 7). Fourteen (73.7%) of 19 patients with yaw modification had severe bony collisions with surgically aberrant configurations of the proximal and distal segments after translation of the 2D treatment plan into 3D simulation, occuring in 46.7% of total cases (14 out of 30) (Table 2).

**Table 1:** Patient information and 2D cephalometry plan for orthognathic surgery.

Pt. No.	Gender/Age (years)	Diagnosis	Surgical procedures 2D plan Position1		
			Le Fort I osteotomy	BSSO	Genioplasty
1	F/32	CP	ANS Adv 3 mm, Impaction R posterior 5 mm, L posterior 2 mm, ML Shift to R 3.5 mm	Setback 5mm	Adv 3mm
2	F/20	BCLP	ANS Adv 5 mm, impaction level U6 2 mm	Setback R 7mm, L 4mm	Adv 6 mm
3	F/17	LCLP	ANS Adv 5 mm, Extrusion 3 mm at U1, 5 mm at LU6, ML Shift to L 3 mm	Setback R 3mm	Nil
4	F/17	BCLP	ANS Adv 10mm Impaction at U1 2mm	Setback R 6 mm, L 4 mm, Kole Setback 8mm	Nil
5	F/18	RCLP	U1 Extrusion 2 mm, PNS Impaction 3mm, ML Shift to L 2 mm	Setback R 10 mm, L 6 mm	Nil
6	M/18	RCLP	2-piece Le Fort I, ANS Adv 10 mm, Extrusion 4mm R U6: Extrusion 4mm, Adv 13mm, L U6: Intrusion 4mm, Adv 7mm ML Shift L 7mm	Setback R 1 mm, L 5mm	Nil
7	M/20	LCLP	ANS Adv 6 mm, R U6 impaction 1mm, Adv 5.5mm; L U6 Adv 4.5mm, ML Shift to L 1 mm	Setback, R 5 mm, L 1 mm	Nil
8	M/18	LCLP	ANS Adv 6 mm, Extrusion 1mm, R U6 impaction 3.5mm, Adv 6mm, L U6 Impaction 0.5mm, Adv 6mm	Setback 5mm	Adv 3mm
9	F/17	LCLP	ANS Adv 4mm, UR6 Impaction 4mm, Adv 2mm, UL6 Impaction 1mm, Adv 5mm	Setback, R 6mm, L 3mm	Shift to R 3 mm
10	F/17	CP	ANS Impaction 7mm, R U6 Adv 9mm, L U6 Adv 11mm	Setback 5mm	Adv 6mm
11	F/15	RCLP	ANS Adv 5mm, U6 level Intrusion 5mm	Setback, R 3mm, L 4mm	Nil
12	M/19	LCLP	ANS Adv 5mm	Setback, R 10mm, L 0mm	Nil
13	M/20	LCLP	Adv 6mm	Setback R 10mm L 8mm	Nil
14	M/19	BCLP	ANS Adv 5mm, Extrusion 1mm, UR6 Impaction 2mm, Adv 2mm, L U6 Impaction 1mm, forward 2mm, ML Shift to R 1mm	Setback R 10 mm, L 17mm	Nil
15	M/18	LCLP	ANS Adv 7mm, R U6 Adv 3mm, L U6 Adv 10mm.	Setback 5mm	Nil
16	M/18	CP	2-piece Le Fort I, R segment Adv 2mm, L segment Adv 6mm, L U6 segment impaction 4mm	Setback 4mm	Adv 3mm, Impaction
17	M/19	LCLP	R U6 Intrusion 4mm	Setback R 10mm, L 8mm	Nil
18	M/18	LCLP	2-piece Le Fort I, ANS Adv 9mm Rotate to right side 3mm, L post segment arch constriction 3mm on L U6	Setback R 6mm, L 3mm	Adv
19	M/19	LCLP	ANS Adv 8mm, Extrusion 3mm	Setback, R 6mm, L 3mm	Nil
20	F/15	LCLP	Adv 5mm	Setback, R 6mm, L 3mm	Nil
21	F/20	BCLP	ANS Adv 5mm, Intrusion 3mm	Setback R 5mm, L 3mm	Adv 6mm
22	M/18	LCLP	ANS Adv 6mm	Setback R 3mm, L 2mm	Nil
23	F/16	RCLP	ANS Adv 5mm, PNS Intrusion 8mm	R Setback 2mm, L Adv 2mm	Nil
24	M/18	LCLP	ANS Adv 8mm, U1 Extrusion 3mm, PNS Impaction 4mm	Setback 5mm	Adv 5mm
25	F/18	CP	ANS Adv 5mm, R U6Adv 8mm Impaction 4mm L U6 Adv 3.5mm	Setback 5mm	Adv
26	M/18	LCLP	2 piece Le Fort I, ANS Adv 8mm, R U6 Extrusion 4mm, L piece Adv 8mm, constriction 8mm on L U6	Setback R 4mm, L 1mm	Nil
27	M/19	BCLP	2-piece Le Fort I, R post segment Adv 3mm, L post segment Adv 5mm, PNS Intrusion 3mm	Setback, R 5mm, L 15mm	Adv. 10mm
28	M/18	LCLP	Adv 10mm, Setback R 9mm, PNS Impaction 3.5mm	Setback, R 9mm, L 7mm	Adv 6mm
29	F/18	BCLP	Adv 8mm, PNS Impaction 4mm, R U6 extrusion 2mm	Setback 3mm	Nil
30	M/20	LCLP	2-piece Le Fort 1, ANS Adv 7mm, R U6 Adv 7mm L U6 Adv 9mm, Intrusion 2mm at U1, Extrusion 3mm at U6 level	Setback 6mm	Adv 5mm

Abbreviations: Pt. No., Patient number; F, female; M, male; LCLP, left cleft lip and palate; RCLP, right cleft lip and palate; BCLP, bilateral cleft lip and palate; CP, cleft palate; BSSO, bilateral sagittal split osteotomy; R, right; L, left; Adv, advancement; U1, central incisor midpoint; U6, upper first molar; ANS, anterior nasal spine; PNS, posterior nasal spine; ML, Midline; SBCC, severe bony collision correction

**Table 2:** Parameter changes from 2D planning to 3D simulation in the CL/P subgroups.

Case No.	Diagnosis	Gender	Age (years)	Yaw (°)	SBC	Roll (°)	Pitch (°)	Mid (mm)	Genio (mm)	No. of PC
3	LCLP	F	17	4	1	5	0	0	0	2
7	LCLP	M	20	0	0	0	0	0	0	0
8	LCLP	M	18	0	0	0	0	1	0	1
9	LCLP	F	17	3	1	3	0	0	0	2
12	LCLP	M	19	0	0	0	0	0	0	0
13	LCLP	M	20	0	0	0	0	0	0	0
15	LCLP	M	18	0	0	0	0	0	0	0
17	LCLP	M	19	0	0	0	0	3	0	1
18	LCLP	M	18	6	1	0	0	0	3	2
19	LCLP	M	19	2.5	0	0	0	4	0	2
20	LCLP	F	15	3	1	0	0	5	0	2
22	LCLP	M	18	2	0	3	0	0	0	2
24	LCLP	M	18	0	0	0	4	1	0	2
26	LCLP	M	18	0	0	7.5	0	0	0	1
28	LCLP	M	18	12	1	1	0	6.5	6.5	4
30	LCLP	M	20	0	0	2	2	0	0	2
5	RCLP	F	18	7	1	4	0	0	0	2
6	RCLP	M	18	8	1	0	0	3	0	2
11	RCLP	F	15	0	0	0	3	0	6	2
23	RCLP	F	16	3.5	0	1	0	1	0	3
**Mean ±SD (range)**	**UCLP**		**18.0±1.4 (15–20)**	**2.6±3.4 (0 to 12)**	**0.4±0.5**	**1.3±2.1 (0–7.5)**	**0.5±1.1 (0–4)**	**1.2±2.0 (0–6.5)**	**0.8±2.0 (0–6.5)**	**1.6±1.0 (0–4)**
2	BCLP	F	20	2	1	0	0	0	0	2
4	BCLP	F	17	2	1	0	0	0	0	1
14	BCLP	M	19	6	1	0	0	0	0	1
21	BCLP	F	20	0	0	0	0	0	0	0
27	BCLP	M	19	7	1	0	0	0	5	2
29	BCLP	F	18	2	0	0	0	0	0	1
**Mean ±SD (range)**	**BCLP**		**18.8±1.2 (17–20)**	**3.2±2.7 (0–7)**	**0.7±0.5**	**0**	**0**	**0.3±0.8 (0–2)**	**0.8±2.0 (0–5)**	**1.2±0.8 (0–2)**
1	CP	F	32	6	1	0	0	0,5	0	2
10	CP	F	17	6	1	2	0	0	0	2
16	CP	M	18	2	0	5	0	2,5	3	4
25	CP	F	18	5	1	0	0	0	6	2
**Mean ±SD (range)**	**CP**		**21.3±7.2 (17–32)**	**4.8±1.9 (2–6)**	**0.8±0.5**	**1.8±2.4 (0–5)**	**0**	**0.8±1.2 (0–2.5)**	**2.3±2.9 (0–6)**	**2.5±1.0 (2–4)**
**Mean ± SD (range)**	**Total**		**18.6±2.9 (15–32)**	**3.0±3.1 (0–12)**	**0.5±0.5**	**1.1±2.0 (0–7.5)**	**0.3±1.0 (0–4)**	**1.0±1.7 (0–6.5)**	**1.0±2.1 (0–6.5)**	**1.6±1.0 (0–4)**

Abbreviations: LCLP: left cleft lip/palate, RCLP: right cleft lip/palate; UCL/P: unilateral cleft lip/palate; BCLP: bilateral cleft lip/palate; CP: isolated cleft palate; M: male, F: female; Age/y, patient age in years; yaw, absolute yaw correction in degrees, SBC, severe bony collision (1: present, 0: not present); roll, absolute roll correction in degrees; pitch, absolute pitch correction in degrees; Mid, midline correction in millimeters; genio, genioplasty alternation in millimeters; No. of PC, number of parameter changes from 2D to 3D plan; SD, standard deviation.

Subgroup analysis of the unilateral CL/P group yielded yaw adjustments in 10 patients (50%, mean 2.6±3.4°, range 0 to 12°) with 7 (35%) patients showing severe bony collisions, roll adjustments in 8 patients (40%, mean 1.3±2.1°, range 0–7.5°), pitch adjustments in 3 patients (15%, mean 0.4±1.1°, range 0–4), midline corrections in 8 patients (40%, mean 1.2±2.0mm, range 0-6-5mm), and genioplasty alterations in 3 patients (15%, mean 0.8±2.0 mm, range 0–6.5mm), with a mean of 1.6±1.0 (range 0–3) parameter changes. The bilateral CL/P subgroup demonstrated yaw correction in all 6 patients (100%, mean 3.2±2.7°, range 0–7°) with severe bony collisions in 4 patients (66%), no roll or pitch corrections, one midline correction of 2mm (16.7%, mean 0.3±0.8mm), and one genioplasty correction of 5 mm (16.7%, mean 0.8±2.0mm) with a mean of 1.2±0.8 parameter changes (range 0–2). The isolated cleft palate subgroup demonstrated yaw correction in all 4 patients (100%, mean 4.8±1.9°, range 2–6°) with severe bony collisions in 3 patients (75%), roll adjustments in 2 patients (50%, mean 1.8±2.4°, range 0–5°), no pitch corrections, midline corrections in 2 patients (50%, mean 0.8±1.2mm, range 0–2.5mm), and genioplasty alterations in 2 patients (50%, mean 2.3±2.9mm, range 0–6mm) with a mean of 2.5±1.0 (range 2–4) parameter changes.

Additionally, post-treatment OQLQ scores were obtained for 20 patients (13 unilateral CL/P, 4 bilateral CL/P, 3 isolated cleft palate) who finished orthodontic treatment (Table 3). Total mean scores were 25.2±17.1, ranging from 2 to 60. Regarding subitem scores, the total mean facial aesthetic score was 7.5±5.0 (range 1–17), the total mean oral function score 3.0±3.6 (range 0–12), the total mean dentofacial aesthetics awareness score 5.0±3.3, (range 0–11), and the total mean score for social aspects of dentofacial deformity 9.8±7.8 (range 0–26). The mean subitem scores for each subgroup (uni-/bilateral CL/P, isolated cleft palate) are shown in Table 3.

**Table 3:** Total and subitem OQLQ scores in the unilateral cleft lip/palate, bilateral cleft lip palate and isolated cleft palate group.

Case No.	Diagnosis	Gender	OQLQ (0–88)	Facial aesthetics (0–20)	Oral Function (0–20)	Awareness (0–16)	Social Aspects (0–32)
7	LCLP	M	29	9	4	8	8
8	LCLP	M	4	1	1	2	0
12	LCLP	M	55	15	7	9	24
13	LCLP	M	5	1	0	0	4
18	LCLP	M	23	7	2	5	9
19	LCLP	M	26	7	4	9	6
20	LCLP	F	12	4	0	4	4
22	LCLP	M	15	2	0	4	9
28	LCLP	M	22	10	4	3	5
5	RCLP	F	11	4	0	3	4
6	RCLP	M	35	15	1	8	11
11	RCLP	F	17	4	0	2	11
23	RCLP	F	10	8	0	0	2
**Mean ±SD (range)**	**UCLP**		**20.3±14.0 (5–55)**	**6.7±4.7 (1–15)**	**1.8±2.3 (0–7)**	**4.4±3.2 (0–9)**	**7.5±6.0 (0–24)**
2	BLCP	F	26	9	6	6	5
14	BLCP	M	2	2	0	0	0
21	BCLP	F	27	3	1	11	12
29	BCLP	F	33	5	6	7	15
**Mean ±SD (range)**	**BCLP**		**22±13.7 (2–33)**	**4.8±3.1 (2–9)**	**3.3±3.2 (0–6)**	**6±4.5 (0–11)**	**8±6.8 (0–15)**
10	CP	F	60	17	10	7	26
16	CP	M	34	13	1	4	16
25	CP	F	57	13	12	8	24
**Mean ±SD (range)**	**CP**		**50.3±14.2 (34–57)**	**14.3±2.3 (13–17)**	**7.7±5.9 (1–12)**	**6.3±2.1 (4–8)**	**22±5.3 (16–26)**
**Mean±SD (range)**	**Total**		**25.2±17.1 (2–60)**	**7.5±5.0 (1–17)**	**3.0±3.6 (0–12)**	**52±3.3 (0–11)**	**9.82±7.8 (0–26)**

Abbreviations: LCLP: left cleft lip/palate, RCLP: right cleft lip/palate, UCLP: unilateral cleft lip/palate, M: male, F: female; OQLQ: Orthognathic quality of life questionnaire; SD: standard deviation

## Discussion

Regarding our primary outcome measure, we can report that in our patient group the large majority (83.3%) of transferred 2D planning datasets had to be adjusted in 3D simulation. Yaw rotation was the most frequently altered parameter and was adjusted in 19 (63.3%) out of 30 patients with the highest mean value and value range. Furthermore, we encountered severe bony collisions (e.g. Fig 3E–3H) in 14 (73.7%) out of 19 patients requiring yaw correction. It is technically difficult to follow such a plan during surgery if anatomical and functional mandibular repositioning is the goal. Following such a flawed plan would result in significant cheek asymmetry or temporo-mandibular joint problems. These issues can be avoided by using 3D simulation, because osseous collisions between the distal and proximal mandibular segments can be addressed in the planning phase and compensated by yaw rotation. Cheek symmetry can furthermore be improved through proper positioning of the proximal segments in the ramus area. Although the maxilla can show antero-posterior discrepancy from both sides in the piriform area after MMC yaw rotation, this asymmetry is comparatively less noticeable and can be corrected by bone grafting or fat injection to the underprojected side. Also, minor changes of the MMC position can conceal any residual asymmetry of the soft tissue [31].

Patients needing midline correction, a total 11 (36.7%) out of 30, were divided in 3 subgroups (Fig 7). In 45.5% of patients the midline was corrected for more than 2mm, in another 45.5% for 1–2mm and in 1 patient (9%) for less than 1mm. Crowded or absent upper incisor dentition can potentially complicate dental midline definition in 2D cephalograms due to the bony and dental overlap. The difference between midline definitions overall and especially between 2D and 3D images can also be a reason for correction [32]. The 3D midline is a plane perpendicular to the Frankfurt horizontal plane through the nasion and basion, whereas the 2D midline is usually the dorsum midline between crista galli, anterior nasal spine, and dental as well as mandibular midpoints [33]. 2D frontal cephalometry distortion and absence of an additional reference point in the skull base may lead to this discrepancy in midline position assessment [34]. Therefore we attribute midline adjustment to a greater information yield of 3D imaging. Especially when combined with roll and yaw correction, the midline can also be altered by rotation in the frontal view [35] and may have to be readjusted. Similar considerations lead to pitch and genioplasty position adjustments. While 2D methods rely on the assumption that the patient’s skull is symmetric, structure superimposition creates distortion that can only be resolved by reassessment in three dimensions [36]. This distortion can be especially detrimental to surgical accuracy in CL/P patients with substantial facial asymmetry.

It must be noted that the case numbers in the subgroups are too small to generalize our findings. However, when looking at the differences and similarities between the subgroups several of the tendencies in our dataset uphold our arguments for 3D simulation advantages over conventional 2D methods. The unilateral CL/P group showed yaw adjustments in 50% and severe bony collisions in 35% of patients; in contrast, all bilateral and isolated cleft palate cases had yaw adjustments, also to a higher mean and range extent. While facial asymmetry is generally believed to be more prominent in unilateral cases [37], these findings could implicate that skeletal facial asymmetry may be underappreciated in bilateral CL/P and isolated cleft palate patients and cannot be detected reliably by 2D methods. Roll correction does not seem to be an issue in bilateral cases, while it is commonly corrected in our unilateral subgroup (40%). Pitch correction is rarely altered compared to 2D cephalometry, with changes in only 15% of our unilateral CL/P patients, and no changes in both our bilateral CL/P and isolated cleft palate patients. Midline adjustments are more apparent and regularly encountered in the unilateral subgroup (40%), but the case number in the bilateral CL/P and isolated cleft palate patients is too limited to extract statistically significant values. Genioplasty alterations can be found in all subgroups; however, positioning the genioplasty segment is the last step in achieving facial symmetry and compensates for the midfacial alterations in the lower part of the face, where asymmetry is most apparent [38]. Therefore, we consider genioplasty alterations to be the result of the previous changes of yaw, roll, pitch and midline and not characteristic for a certain subgroup.

**Fig 8:**
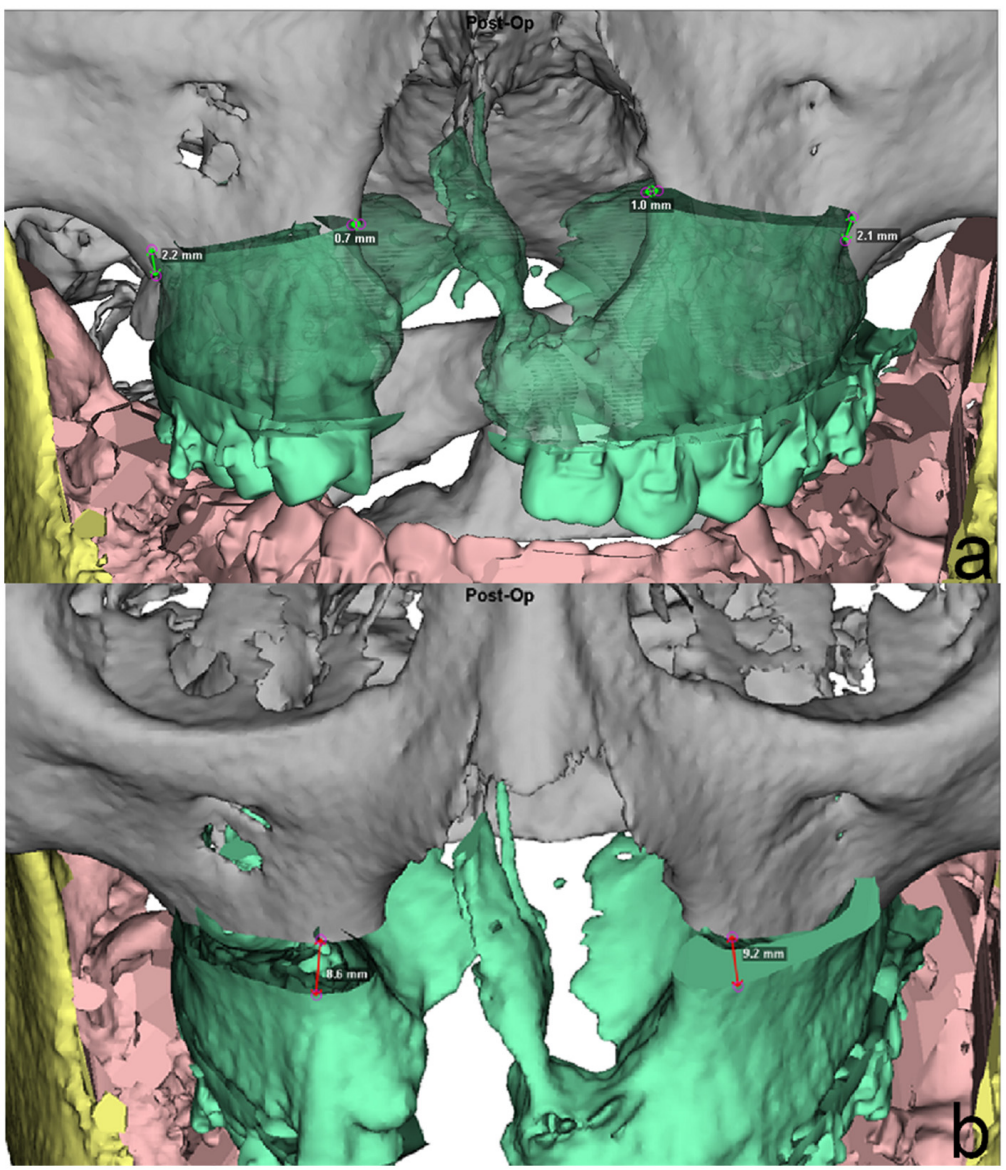
Position analysis of the Le Fort I segment of sample patient #29 (Table 1). Bilateral cleft lip/palate patient. The 3D position of the Le Fort I segment in relation to the superior fixed part of the maxilla is recorded in both lateral and piriform areas to facilitate proper positioning of the MMC during surgery. Midline shifts in the patient’s right lateral pillar and impaction of the left piriform aperture (Fig 5a) are noted as well as the different amounts of advancement or setback in both left and right lateral and piriform levels (Fig 5b). This information is the result of the 3D alternation of the MMC and helps to transfer the results of 3D simulation into the surgical setting.

The impact of 3D planning during single-splint surgery is manifold. The exact Le Fort I segment position can be evaluated preoperatively (Fig 8). The 3D position of the Le Fort I segment in relation to the maxilla is recorded in both lateral and piriform areas to facilitate proper positioning of the MMC during surgery. Midline shift, in this case to the right, and impaction in both upper first molar and piriform levels (Fig 8a) are noted as well as the different amounts of advancement or setback in the aforemetioned four levels (Fig 8b). The Le Fort I to maxilla relation defines the overall MMC position and can then be transferred by CAD/CAM manufactured splints, intraoperative navigation and/or positioning guides. Numerous studies have shown the higher accuracy of 3D simulation compared to traditional 2D planning [10, 39]. A recent randomized controlled trial showed that alignment of the lower inter-incisal point (p = 0.03), mandibular sagittal plane (p = 0.01), and centering of the dental midlines (p = 0.03) were significantly more accurate in patients with 3D simulation than in the traditional 2D group [12]. For we also transfer our planning information with CAD/CAM splints, we can clinically confirm previous findings of a high-degree correlation between predicted and actual outcomes of 3D planning [9]. A prospective multicenter study demonstrated a maximum root-mean-square difference in pre- and postoperative CBCT images of 1.0 mm and 1.5 degrees for the maxilla and 1.1 mm and 1.8 degrees for the mandible [11]. Our group also published that MMC position can be reproduced during surgery by either intraoperative navigation [40] with a mean accuracy of 0.66 mm difference (range 0.05–1.46mm), or positioning guides [39] with accuracies of 0.18–0.33mm in the maxilla and 0.99–1.56mm in the mandible comparing the superimposed pre- and postoperative CBCT images [17].

Increased freedom of movement in single-splint OGS also leads to a longer learning period, making the visualization of the exact Le Fort I segment position especially useful. MMC control can be challenging for the beginner and sometimes even for the experienced surgeon, more so if the preoperative evaluation is insufficient. In contrast to 2D cephalometry, 3D simulation also facilitates thorough morphology analysis and helps to reduce the surgical difficulty by predicting problems like bony collisions and anomalies. The level and type of Le Fort I osteotomy can be assessed in more detail. Planning adjustments (e.g. two-segmental Le Fort I) after failed alveolar bone grafting, collisions in the pterygoid area after yaw rotation or setback, and the level of the Le Fort I osteotomy for correcting under-projection of the piriform area on the cleft side can also be anticipated.

Drawbacks of 2D planning for mandibular positioning show up in 3D simulation after treatment plan transfer. Osseous collisions or gaps in the BSSO segments and the need of bone grafting are reliably detected in 3D simulation [41], making the ramus area adjustments predictable in terms of lower face symmetry. This finding is regularly missed in 2D cephalograms (Figs 3H and 4H), but easily corrected in the 3D basal view (Figs 3L and 4L). Moreover, the course of the inferior alveolar nerve in the mandibular body can be visualized preoperatively, making the BSSO procedure safer and more predictable [42].

Advocates of traditional planning methods argue that jaw and dental relations can be precisely simulated without the use of 3D methods. However, a recent investigation shows that face-bow transfer accuracy is inferior to 3D simulation in terms of anatomical reference plane definition [43]. While stone model casts are still needed for digitalization to ensure proper representation of the occlusal surface in the CBCT pictures [26], the remaining steps can easily be done in 3D simulation, and the development of intraoral scanners is already promoted to overcome these limitations and completely digitalize the data acquisition process [44]. Several options of MMC placement can be evaluated during 3D simulation without the need of going through repeated traditional articulator setups. It is a general trend that 3D simulation is about to replace traditional analytical model surgery based on 2D treatment plans [45], especially when two-jaw OGS is conducted [46]. Also, the traditional stone model method mainly focuses on occlusal relation and does not address overall facial harmony, because the soft tissue envelope cannot be assessed.

We can additionally report that the post-treatment evaluation of OQLQ scores showed comparable scores to patients in a previously published non-cleft control group, and largely higher scores than patients in the preoperative group of non-cleft patients [30]. Dental function was assessed very positive among all the subgroups. In our patient sample, the unilateral CL/P group showed the highest overall satisfaction with a close following by the bilateral CL/P group. While we cannot report a significant case number in the isolated cleft palate group, these patients seem to have a tendency for a more impaired quality of life and especially a larger burden regarding social aspects of the dentofacial deformity compared to the cleft lip groups. However, these findings are purely descriptive due to the limited case number and warrant further evaluation in a separate study.

Limitations of our study include the focus on planning and conducting two-jaw OGS solely for cleft lip/palate patients. Also, our subgroup analysis can only be descriptive in both functional and outcome measures due to the limited case number, and conclusions should not be generalized. However, certain tendencies can be recognized in the data, and while the planning responsibilities and sequences are diverse in OGS practice around the world, there is still applicable information from this study regardless of these differences. Firstly, the surgeon has to be aware that the intraoperative situation is less predictable and the accurate transfer of the plan may be harder to achieve with a 2D cephalometry-based surgical plan. Reaching a treatment consensus is much easier if both orthodontist and surgeon can adjust their planning together using a 3D simulation that is based on more overall information. Secondly, while we conduct our surgery using only the single-splint and not the two-splint method, both techniques need proper preoperative planning. 3D planning and CAD/CAM splint fabrication for two-splint surgery is already being carried out with high accuracy [44], and addressing bony collisions in the ramus area beforehand is even more useful in two-splint surgery, possibly avoiding the need for intraoperative adjustments and losing the purpose of the intermediate occlusal splint altogether. Finally, our patient collection is limited to CL/P patients, where more prominent features of facial asymmetry may translate into a higher incidence of treatment plan alterations; however, in our experience we regularly alter treatment plans of non-cleft patients with facial asymmetry and also frequently observe severe bony collisions in the ramus area. Therefore we are currently preparing another study focusing on the incidence of same treatment plan changes in the general population. The subgroup analysis of unilateral CL/P, bilateral CL/P and isolated cleft palate treatment plan adjustments should also be expanded in further subgroup studies to validate the trends we have seen in this investigation.

## Conclusion

In our patient group, consisting exclusively of CL/P patients, we demonstrated the greater information yield of 3D simulation over 2D planning. 83.3% of our 2D surgical plans were modified, mostly concerning yaw and midline adjustments. Severe bony collisions, occurring in a total 46.7% of cases, could regularly be anticipated and corrected during 3D simulation before surgery. Based on our findings, 3D simulation renders important information for accurate planning in complex CL/P cases involving facial asymmetry that is regularly missed in 2D methods. Intraoperative findings can be predicted due to more detailed imaging of the facial bony structures in terms of position, orientation and form, and major intraoperative changes of the treatment plan due to lack of technical feasibility can be avoided.

## References

[1] Proffit WR, Fields HW Jr, Moray LJ. Prevalence of malocclusion and orthodontic treatment need in the United States: estimates from the NHANES III survey. The International journal of adult orthodontics and orthognathic surgery. 1998;13(2):97–106. Epub 1998/09/23. . 9743642

[2] Altobelli DE, Kikinis R, Mulliken JB, Cline H, Lorensen W, Jolesz F. Computer-assisted three-dimensional planning in craniofacial surgery. Plastic and reconstructive surgery. 1993;92(4):576–85; discussion 86–7. Epub 1993/09/01. . 8356120

[3] Caloss R, Atkins K, Stella JP. Three-dimensional imaging for virtual assessment and treatment simulation in orthognathic surgery. Oral and maxillofacial surgery clinics of North America. 2007;19(3):287–309, v. Epub 2007/12/20. 10.1016/j.coms.2007.04.006 . 18088886

[4] Haas OL Jr, Becker OE, de Oliveira RB. Computer-aided planning in orthognathic surgery-systematic review. International journal of oral and maxillofacial surgery. 2014 Epub 2014/11/30. 10.1016/j.ijom.2014.10.025 . 25432508

[5] Kusnoto B. Two-dimensional cephalometry and computerized orthognathic surgical treatment planning. Clinics in plastic surgery. 2007;34(3):417–26. Epub 2007/08/21. 10.1016/j.cps.2007.04.005 . 17692701

[6] da Silva MB, Sant'Anna EF. The evolution of cephalometric diagnosis in orthodontics. Dental press journal of orthodontics. 2013;18(3):63–71. Epub 2013/10/08. . 24094013 10.1590/s2176-94512013000300011

[7] Gateno J, Xia JJ, Teichgraeber JF, Christensen AM, Lemoine JJ, Liebschner MA, et al Clinical feasibility of computer-aided surgical simulation (CASS) in the treatment of complex cranio-maxillofacial deformities. Journal of oral and maxillofacial surgery: official journal of the American Association of Oral and Maxillofacial Surgeons. 2007;65(4):728–34. Epub 2007/03/21. 10.1016/j.joms.2006.04.001 . 17368370

[8] Xia JJ, Gateno J, Teichgraeber JF, Christensen AM, Lasky RE, Lemoine JJ, et al Accuracy of the computer-aided surgical simulation (CASS) system in the treatment of patients with complex craniomaxillofacial deformity: A pilot study. Journal of oral and maxillofacial surgery: official journal of the American Association of Oral and Maxillofacial Surgeons. 2007;65(2):248–54. Epub 2007/01/24. 10.1016/j.joms.2006.10.005 . 17236929

[9] Aboul-Hosn Centenero S, Hernandez-Alfaro F. 3D planning in orthognathic surgery: CAD/CAM surgical splints and prediction of the soft and hard tissues results—our experience in 16 cases. Journal of cranio-maxillo-facial surgery: official publication of the European Association for Cranio-Maxillo-Facial Surgery. 2012;40(2):162–8. Epub 2011/04/05. 10.1016/j.jcms.2011.03.014 . 21458285

[10] Lin HH, Lo LJ. Three-dimensional computer-assisted surgical simulation and intraoperative navigation in orthognathic surgery: a literature review. Journal of the Formosan Medical Association = Taiwan yi zhi. 2015;114(4):300–7. Epub 2015/03/07. 10.1016/j.jfma.2015.01.017 . 25744942

[11] Hsu SS, Gateno J, Bell RB, Hirsch DL, Markiewicz MR, Teichgraeber JF, et al Accuracy of a computer-aided surgical simulation protocol for orthognathic surgery: a prospective multicenter study. Journal of oral and maxillofacial surgery: official journal of the American Association of Oral and Maxillofacial Surgeons. 2013;71(1):128–42. Epub 2012/06/15. 10.1016/j.joms.2012.03.027 22695016 PMC3443525

[12] De Riu G, Meloni SM, Baj A, Corda A, Soma D, Tullio A. Computer-assisted orthognathic surgery for correction of facial asymmetry: results of a randomised controlled clinical trial. The British journal of oral & maxillofacial surgery. 2014;52(3):251–7. Epub 2014/01/15. 10.1016/j.bjoms.2013.12.010 . 24418178

[13] Li B, Zhang L, Sun H, Yuan J, Shen SG, Wang X. A novel method of computer aided orthognathic surgery using individual CAD/CAM templates: a combination of osteotomy and repositioning guides. The British journal of oral & maxillofacial surgery. 2013;51(8):e239–44. Epub 2013/04/10. 10.1016/j.bjoms.2013.03.007 . 23566536

[14] Shehab MF, Barakat AA, AbdElghany K, Mostafa Y, Baur DA. A novel design of a computer-generated splint for vertical repositioning of the maxilla after Le Fort I osteotomy. Oral Surg Oral Med Oral Pathol Oral Radiol. 2013;115(2):e16–25. Epub 2013/01/15. 10.1016/j.oooo.2011.09.035 . 23312923

[15] Sun Y, Luebbers HT, Agbaje JO, Schepers S, Vrielinck L, Lambrichts I, et al Accuracy of upper jaw positioning with intermediate splint fabrication after virtual planning in bimaxillary orthognathic surgery. The Journal of craniofacial surgery. 2013;24(6):1871–6. Epub 2013/11/14. 10.1097/SCS.0b013e31829a80d9 . 24220365

[16] Zinser MJ, Sailer HF, Ritter L, Braumann B, Maegele M, Zoller JE. A paradigm shift in orthognathic surgery? A comparison of navigation, computer-aided designed/computer-aided manufactured splints, and "classic" intermaxillary splints to surgical transfer of virtual orthognathic planning. Journal of oral and maxillofacial surgery: official journal of the American Association of Oral and Maxillofacial Surgeons. 2013;71(12):2151.e1–21. Epub 2013/11/19. 10.1016/j.joms.2013.07.007 . 24237776

[17] Lin HH, Chang HW, Wang CH, Kim SG, Lo LJ. Three-dimensional computer-assisted orthognathic surgery: experience of 37 patients. Annals of plastic surgery. 2015;74 Suppl 2:S118–26. Epub 2015/03/19. 10.1097/sap.0000000000000455 . 25785379

[18] Xia JJ, Shevchenko L, Gateno J, Teichgraeber JF, Taylor TD, Lasky RE, et al Outcome study of computer-aided surgical simulation in the treatment of patients with craniomaxillofacial deformities. Journal of oral and maxillofacial surgery: official journal of the American Association of Oral and Maxillofacial Surgeons. 2011;69(7):2014–24. Epub 2011/06/21. 10.1016/j.joms.2011.02.018 ; PubMed Central PMCID: PMCPmc3119456. 21684451 PMC3119456

[19] Gateno J, Xia JJ, Teichgraeber JF. New 3-dimensional cephalometric analysis for orthognathic surgery. Journal of oral and maxillofacial surgery: official journal of the American Association of Oral and Maxillofacial Surgeons. 2011;69(3):606–22. Epub 2011/01/25. 10.1016/j.joms.2010.09.010 ; PubMed Central PMCID: PMCPmc3059215. 21257250 PMC3059215

[20] Kwon TG, Park HS, Ryoo HM, Lee SH. A comparison of craniofacial morphology in patients with and without facial asymmetry—a three-dimensional analysis with computed tomography. International journal of oral and maxillofacial surgery. 2006;35(1):43–8. Epub 2005/06/01. . 15925488 10.1016/j.ijom.2005.04.006

[21] Farkas LG, Cheung G. Facial asymmetry in healthy North American Caucasians. An anthropometrical study. The Angle orthodontist. 1981;51(1):70–7. Epub 1981/01/01. . 6939355 10.1043/0003-3219(1981)051<0070:FAIHNA>2.0.CO;2

[22] Abuhijleh E, Aydemir H, Toygar-Memikoglu U. Three-dimensional craniofacial morphology in unilateral cleft lip and palate. Journal of oral science. 2014;56(2):165–72. Epub 2014/06/17. . 24930754 10.2334/josnusd.56.165

[23] Meyer-Marcotty P, Alpers GW, Gerdes AB, Stellzig-Eisenhauer A. Impact of facial asymmetry in visual perception: a 3-dimensional data analysis. American journal of orthodontics and dentofacial orthopedics: official publication of the American Association of Orthodontists, its constituent societies, and the American Board of Orthodontics. 2010;137(2):168.e1–8; discussion -9. Epub 2010/02/16. 10.1016/j.ajodo.2008.11.023 . 20152669

[24] Rinchuse DJ, Kandasamy S. Articulators in orthodontics: an evidence-based perspective. American journal of orthodontics and dentofacial orthopedics: official publication of the American Association of Orthodontists, its constituent societies, and the American Board of Orthodontics. 2006;129(2):299–308. Epub 2006/02/14. 10.1016/j.ajodo.2005.03.019 . 16473725

[25] Dai J, Tang M, Xin P, Hu G, Si J, Dong Y, et al Accurate movement of jaw segment in virtual 3D orthognathic surgery. The Journal of craniofacial surgery. 2014;25(2):e140–3. Epub 2014/03/14. 10.1097/scs.0000000000000414 . 24621754

[26] Lin HH, Chiang WC, Lo LJ, Sheng-Pin Hsu S, Wang CH, Wan SY. Artifact-resistant superimposition of digital dental models and cone-beam computed tomography images. Journal of oral and maxillofacial surgery: official journal of the American Association of Oral and Maxillofacial Surgeons. 2013;71(11):1933–47. Epub 2013/08/06. 10.1016/j.joms.2013.06.199 . 23911142

[27] Lin HH, Chuang YF, Weng JL, Lo LJ. Comparative validity and reproducibility study of various landmark-oriented reference planes in 3-dimensional computed tomographic analysis for patients receiving orthognathic surgery. PloS one. 2015;10(2):e0117604 Epub 2015/02/11. 10.1371/journal.pone.0117604 25668209 PMC4323243

[28] Cunningham SJ, Garratt AM, Hunt NP. Development of a condition-specific quality of life measure for patients with dentofacial deformity: I. Reliability of the instrument. Community dentistry and oral epidemiology. 2000;28(3):195–201. Epub 2000/06/01. . 10830646 10.1034/j.1600-0528.2000.280305.x

[29] Cunningham SJ, Garratt AM, Hunt NP. Development of a condition-specific quality of life measure for patients with dentofacial deformity: II. Validity and responsiveness testing. Community dentistry and oral epidemiology. 2002;30(2):81–90. Epub 2002/05/10. . 12000348 10.1034/j.1600-0528.2002.300201.x

[30] Lee S, McGrath C, Samman N. Impact of orthognathic surgery on quality of life. Journal of oral and maxillofacial surgery: official journal of the American Association of Oral and Maxillofacial Surgeons. 2008;66(6):1194–9. Epub 2008/05/20. 10.1016/j.joms.2008.01.006 . 18486784

[31] Bergeron L, Yu CC, Chen YR. Single-splint technique for correction of severe facial asymmetry: correlation between intraoperative maxillomandibular complex roll and restoration of mouth symmetry. Plastic and reconstructive surgery. 2008;122(5):1535–41. Epub 2008/10/31. 10.1097/PRS.0b013e31818820d8 . 18971738

[32] Maglione M, Costantinides F. Localization of basicranium midline by submentovertex projection for the evaluation of condylar asymmetry. International journal of dentistry. 2012;2012:285693 Epub 2012/02/09. 10.1155/2012/285693 ; PubMed Central PMCID: PMCPmc3272349. 22315603 PMC3272349

[33] Hurst CA, Eppley BL, Havlik RJ, Sadove AM. Surgical cephalometrics: applications and developments. Plastic and reconstructive surgery. 2007;120(6):92e–104e. Epub 2007/11/28. . 18040171 10.1097/01.prs.0000282728.97278.a2

[34] Chien PC, Parks ET, Eraso F, Hartsfield JK, Roberts WE, Ofner S. Comparison of reliability in anatomical landmark identification using two-dimensional digital cephalometrics and three-dimensional cone beam computed tomography in vivo. Dento maxillo facial radiology. 2009;38(5):262–73. Epub 2009/05/29. 10.1259/dmfr/81889955 . 19474253

[35] Song WW, Kim SS, Sandor GK, Kim YD. Maxillary yaw as the primary predictor of maxillary dental midline deviation: 3D analysis using cone-beam computed tomography. Journal of oral and maxillofacial surgery: official journal of the American Association of Oral and Maxillofacial Surgeons. 2013;71(4):752–62. Epub 2012/09/27. 10.1016/j.joms.2012.07.043 . 23010372

[36] Halazonetis DJ. From 2-dimensional cephalograms to 3-dimensional computed tomography scans. American journal of orthodontics and dentofacial orthopedics: official publication of the American Association of Orthodontists, its constituent societies, and the American Board of Orthodontics. 2005;127(5):627–37. Epub 2005/05/07. 10.1016/j.ajodo.2005.01.004 . 15877045

[37] Guyuron B. MOC-PS(SM) CME article: late cleft lip nasal deformity. Plastic and reconstructive surgery. 2008;121(4 Suppl):1–11. Epub 2008/04/11. 10.1097/01.prs.0000305955.67554.40 . 18379379

[38] Huang CS, Liu XQ, Chen YR. Facial asymmetry index in normal young adults. Orthodontics & craniofacial research. 2013;16(2):97–104. Epub 2013/01/18. 10.1111/ocr.12010 . 23324075

[39] Lin HH, Chang HW, Lo LJ. Development of customized positioning guides using computer-aided design and manufacturing technology for orthognathic surgery. International journal of computer assisted radiology and surgery. 2015;10(12):2021–33. Epub 2015/05/20. 10.1007/s11548-015-1223-0 . 25981638

[40] Chang HW, Lin HH, Chortrakarnkij P, Kim SG, Lo LJ. Intraoperative navigation for single-splint two-jaw orthognathic surgery: From model to actual surgery. Journal of cranio-maxillo-facial surgery: official publication of the European Association for Cranio-Maxillo-Facial Surgery. 2015;43(7):1119–26. Epub 2015/07/15. 10.1016/j.jcms.2015.06.009 . 26160383

[41] Mori Y, Shimizu H, Minami K, Kwon TG, Mano T. Development of a simulation system in mandibular orthognathic surgery based on integrated three-dimensional data. Oral and maxillofacial surgery. 2011;15(3):131–8. Epub 2010/10/29. 10.1007/s10006-010-0247-4 ; PubMed Central PMCID: PMCPmc3157604. 20981462 PMC3157604

[42] Huang CS, Syu JJ, Ko EW, Chen YR. Quantitative evaluation of cortical bone thickness in mandibular prognathic patients with neurosensory disturbance after bilateral sagittal split osteotomy. Journal of oral and maxillofacial surgery: official journal of the American Association of Oral and Maxillofacial Surgeons. 2013;71(12):2153.e1–10. Epub 2013/10/19. 10.1016/j.joms.2013.08.004 . 24135253

[43] Zizelmann C, Hammer B, Gellrich NC, Schwestka-Polly R, Rana M, Bucher P. An evaluation of face-bow transfer for the planning of orthognathic surgery. Journal of oral and maxillofacial surgery: official journal of the American Association of Oral and Maxillofacial Surgeons. 2012;70(8):1944–50. Epub 2011/11/15. 10.1016/j.joms.2011.08.025 . 22079061

[44] Hernandez-Alfaro F, Guijarro-Martinez R. New protocol for three-dimensional surgical planning and CAD/CAM splint generation in orthognathic surgery: an in vitro and in vivo study. International journal of oral and maxillofacial surgery. 2013;42(12):1547–56. Epub 2013/06/19. 10.1016/j.ijom.2013.03.025 . 23768749

[45] Lonic D, Lo LJ. Three-dimensional simulation of orthognathic surgery-surgeon's perspective. Journal of the Formosan Medical Association = Taiwan yi zhi. 2015 Epub 2015/10/21. 10.1016/j.jfma.2015.09.002 . 26482093

[46] Hammoudeh JA, Howell LK, Boutros S, Scott MA, Urata MM. Current Status of Surgical Planning for Orthognathic Surgery: Traditional Methods versus 3D Surgical Planning. Plastic and reconstructive surgery Global open. 2015;3(2):e307 Epub 2015/03/10. 10.1097/gox.0000000000000184 25750846 PMC4350313

